# *In Silico* Insight into Potential Anti-Alzheimer’s Disease Mechanisms of Icariin

**DOI:** 10.3390/ijms17010113

**Published:** 2016-01-15

**Authors:** Zhijie Cui, Zhen Sheng, Xinmiao Yan, Zhiwei Cao, Kailin Tang

**Affiliations:** 1 School of Life Sciences and Technology, Tongji University, 1239 Siping Road, Shanghai 200092, China; zjcui@tongji.edu.cn (Z.C.); 1110628@tongji.edu.cn (Z.S.); 1434316@tongji.edu.cn (X.Y.); zwcao@tongji.edu.cn (Z.C.); 2 Advanced Institute of Translational Medicine, Tongji University, 1239 Siping Road, Shanghai 200092, China

**Keywords:** icariin, Alzheimer’s disease (AD), INVDOCK, network pharmacology

## Abstract

Herbal compounds that have notable therapeutic effect upon Alzheimer's disease (AD) have frequently been found, despite the recent failure of late-stage clinical drugs. Icariin, which is isolated from Epimedium brevicornum, is widely reported to exhibit significant anti-AD effects in *in vitro* and *in vivo* studies. However, the molecular mechanism remains thus far unclear. In this work, the anti-AD mechanisms of icariin were investigated at a target network level assisted by an *in silico* target identification program (INVDOCK). The results suggested that the anti-AD effects of icariin may be contributed by: attenuation of hyperphosphorylation of tau protein, anti-inflammation and regulation of Ca^2+^ homeostasis. Our results may provide assistance in understanding the molecular mechanism and further developing icariin into promising anti-AD agents.

## 1. Introduction

Alzheimer’s disease (AD) is known as a common form of dementia and characterized by progressive cognitive deterioration, neuropsychiatric and behavioral symptoms in clinical studies, while its incidence is largely increasing [1]. Until now, there have been mainly two classes of drugs approved by FDA to ameliorate the cognitive problems of AD. One class of drugs target acetylcholinesterase (AChE) [2], including tacrine, donepezil, rivastigmine, galantamine. The others target the N-methyl-d-aspartate receptors (NMDARs) (memantine, *etc.*) [3]. However, these drugs were frequently reported to habe limited effect as they can only relieve the symptoms of AD, instead of stopping or reversing the disease progression [4,5]. Meanwhile, a large number of compounds including natural ingredients from herbs have been screened to meet the urgent demand for new anti-AD drugs, among which a flavonol glycoside, icariin, was frequently shown to have potential anti-AD effects in various studies.

Being derived from Horny Goat Weed, which belongs to the genus *Epimedium*, icariin was first detected in 2009 to inhibit amyloid-beta peptide (Abeta)-induced neurotoxicity by upregulating cocaine-regulated and amphetamine-regulated transcripts (CART) in cortical neuron cells [6]. Then further studies in PC12 cells showed icariin’s protective effects against neurotoxicity through activating PI3K/Akt signaling pathway [7,8], inhibiting phosphorylation of JNK/p38 MAPK and p53 activity [9]. The similar effect was observed in rat hippocampal slice by suppressing the abnormal inward calcium currents [10]. Furthermore, it was demonstrated that icariin could improve learning and memory abilities in AD mice/rats models through suppression of beta-secretase expression [11], attenuation of neurite atrophy [12], stimulation of NO/cGMP signaling and co-ordinated induction of nitric oxide synthase (NOS) isoforms [13]. Notably, it was reported that icariin could inhibit the activity of AChE, which was a main therapeutic target of AD [14].

The above experiments have provided insightful evidence of the promising anti-AD effects for icariin. It is subsequently interesting to investigate the anti-AD mechanism of the compound. Four proteins have been individually identified as interacting with icariin through differential scanning fluorimetry, high performance liquid chromatography (HPLC) and the two-step radioisotope procedure respectively, including acetylcholinesterase (AChE) [14], UGT1A7, UGT1A9 [15] and PDE5 [16]. However, the overall target profile and molecular mechanisms of action (MOA) remain unknown and deserve further investigation. Nowadays, the techniques of system biology have been widely applied to explore the compound-target-disease relationship in a systematic way. An interesting example was set by Sun *et al.* where targets of natural compounds would be used as the molecular probes to suggest the overall network of AD pathogenesis [17]. Their results indicated that targeting multiple pathways of the AD symptom pathway, the inflammation pathway, the cancer pathway, the diabetes mellitus pathway, the intracellular Ca^2+^ homeostasis pathway, and cell proliferation pathway may contribute to the anti-AD effects of the natural compounds. In our study, a target auto-identification program of INVDOCK [18] was employed to calculate the potential target profile for icariin, then the MOA of icariin was suggested in the background of biological regulatory pathways related to anti-AD effects.

## 2. Results

### 2.1. The Putative Protein Targets of Icariin

**Figure 1:**
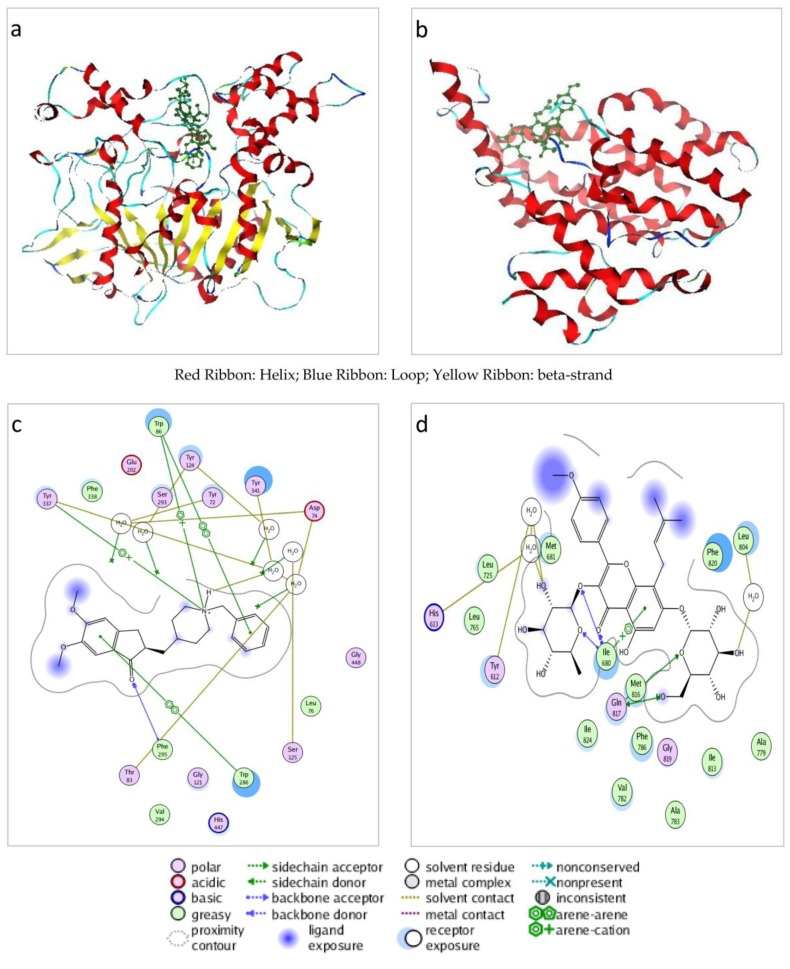
Illustration of icariin docked into acetylcholinesterase (AChE) A chain (**a**,**c**) and PDE5 A chain (**b**,**d**).

A total of 798 neurodegenerative disease-related proteins were obtained with the Protein Data Bank (PDB) cavity structures [18]. The pre-processed 3D structure of icariin was used to search for potential targets among the 798 proteins. 59 distinct proteins were computationally identified as putative targets of icariin. Among these putative targets, 39 are known therapeutic targets targeted by FDA-approved and experimental drugs (Supplementary Materials Table S1). Among the four known proteins interacting with icariin, two proteins (PDE5 and AchE), were included in 798 neurodegenerative disease-related proteins on account of the availability of PDB structures. The two targets were both successfully predicted as putative targets by INVDOCK. The putative complexes of icariin binding with AChE and PDE5 were shown in Figure 1a–d respectively.

As the direct binding targets of icariin were sparsely known in the literature, comparing the binding energy difference between target-icariin and target-drug may give alternative evidence [19]. Among the 59 putative protein targets, 39 proteins (which were known therapeutic targets, targeted by FDA-approved or experimental drugs) were docked by the drug and icariin, respectively. In the process, the PDB complex structure of a target was was prior to be chosen if its native ligand was a corresponding drug of the target. Twenty-one (53.85%) icariin-target interactions showed comparative binding affinities (better or close molecular-mechanics generalized born/volume integral (MM/GBVI) or pki value) to their corresponding target-drug interactions (shown in Table 1). These targets were regarded as experiencing a strong or true effect by icariin, while the remaining 18 (46.15%) would be viewed as “weak” binding targets of icariin (or some of them even might be “false positives”). Notably, these “weak” targets could not be excluded because synergistic effects of multi-targets were often considered in conventional pharmacological studies of herbs [20,21]. All results for comparative docking analysis were listed in Supplementary Materials Table S2. In addition, we provided all interaction pose files in the Table 1 as Supplementary Materials 3.

### 2.2. The Potential Targets Significantly Correlate with AD-Related Proteins

Meanwhile, 89 AD-related proteins (ADPs) were retrieved from the Comparative Toxicogenomics Database (CTD). The functional correlation between the 59 icariin’s putative targets and the 89 ADPs were calculated in both PPI network and Gene Ontology (GO) term similarities. In the Human Protein Reference Database (HPRD) protein-prontein interaction (PPI) network, the average shortest path between 59 putative targets and 89 ADPs turned out to be 3.676. Two randomizations were done separately for either ADPs or putative targets. For each randomization, a group of proteins were randomly picked from the whole human proteins with the same number as the number of ADPs or putative targets. Each randomization process was repeated 1,000,000 times, and the distribution of the average shortest path for random sampling was obtained, respectively. The Z-scores were both above 4 (4.06 and 4.13). Compared with random sampling, the distance between putative targets and ADPs are significantly short in the PPI network, which indicated the close relationship between icariin’s putative targets and ADPs.

**Table 1:** The binding affinity comparison between icariin and corresponding known ligand in the same protein target (strong/true effects). For each therapeutic target, the same active pocket site was adopted for binding affinity comparison between icariin and known ligands. The docking was demonstrated and refined using the Molecular Operating Environment (MOE) with the parameters of Receptor: Receptor + Solvent; Placement: Triangle Mather; Rescoring 1: London dG, Retain: 30; Refinement: Forcefield (MMFF94x); Rescoring 2: London dG, Retain: 30.

Uniprot	Gene Symbol	PDB Code	Known Ligand			Icariin	
			Name	MM/GBVI (kcal/mol)	Affinity (pki)	MM/GBVI (kcal/mol)	Affinity (pki)
Q13464	ROCK1	2ETK	Hydroxyfasudil	−21.03	8.10	−33.03	14.34
P00439	PAH	4PAH	Norepinephrine	−26.24	7.05	−44.04	7.90
Q9HAN9	NMNAT1	1GZU	Nicotinamide Mononucleotide	−27.30	12.54	−23.15	17.75
Q9BW91	NUDT9	1Q33	β-d-Glucose	−24.64	10.70	−34.00	13.26
P50135	HNMT	2AOU	Amodiaquine	−26.05	6.91	−22.14	6.10
Q10588	BST1	1ISG	Adenosine-5′-diphosphate Monothiophosphate	−14.33	9.41	−24.31	10.99
P06737	PYGL	1FA9	Adenosine Monophosphate	−17.06	8.66	−27.18	9.79
P00750	PLAT	1PK2	Aminocaproic Acid	−19.90	9.71	−24.92	9.30
O76074	PDE5	2H42	Sildenafil	−36.25	9.67	−28.87	13.89
P04062	GBA	2F61	2-(Acetylamino)-2-deoxy-a-d-glucopyranose	−16.41	5.57	−21.80	11.17
P15291	B4GALT1	4EEG	*N*-Acetyl-d-glucosamine	−19.15	8.97	−34.21	10.75
P07737	PFN1	1CJF	7-Hydroxy-4-methyl-3-(2-hydroxy-ethyl)coumarin	−15.42	6.69	−27.05	8.67
P09012	SNRPA	1NU4	Malonic Acid	−22.16	6.52	−23.86	5.34
P84077	ARF1	1U81	1,3-Propandiol	−37.77	4.64	−43.17	11.18
Q08209	PPP3CA	4F0Z	Myristic Acid	−15.56	4.64	−22.51	8.90
P13569	CFTR	2BBO	Ibuprofen	−8.23	6.00	−6.62	11.01
P02774	GC	1J78	Cholecalciferol	−10.99	5.05	−21.56	6.43
P11387	TOP1	1TL8	Irinotecan	−34.75	14.32	−20.04	17.80
P19883	FST	2B0U	d-Myo-inositol-hexasulphate	−9.54	7.03	−24.81	9.78
P27695	APEX1	4QHE	Lucanthone	−16.70	4.25	−17.73	4.56
P22303	AChE	1F8U	Mefloquine	−11.44	6.54	−34.86	7.97

In addition, the functional correlation of that two groups of proteins were further measured by the semantic similarity of annotated GO profiles. Fifty nine icariin’s putative targets and 89 ADPs were annotated by two profiles of GO terms, respectively. In this work, each GO term, which referred to biological processes and significantly affected (*p* < 0.05) in level 4, was chosen and added into the corresponding GO profile. The semantic similarity of 59 putative targets and ADPs were calculated to be 0.664. Randomized simulative experiments were similarly employed of 1,000,000 times for either ADPs or putative targets, respectively. It was inferred that the similarity of GO profiles of putative targets and ADPs was significant (*p*-value 0.039 and 0.031). The above results suggested that the predicted icariin’s targets significantly correlate with the AD-related proteins.

### 2.3. An Integrated Network for Anti-AD Effects of Icariin

**Figure 2:**
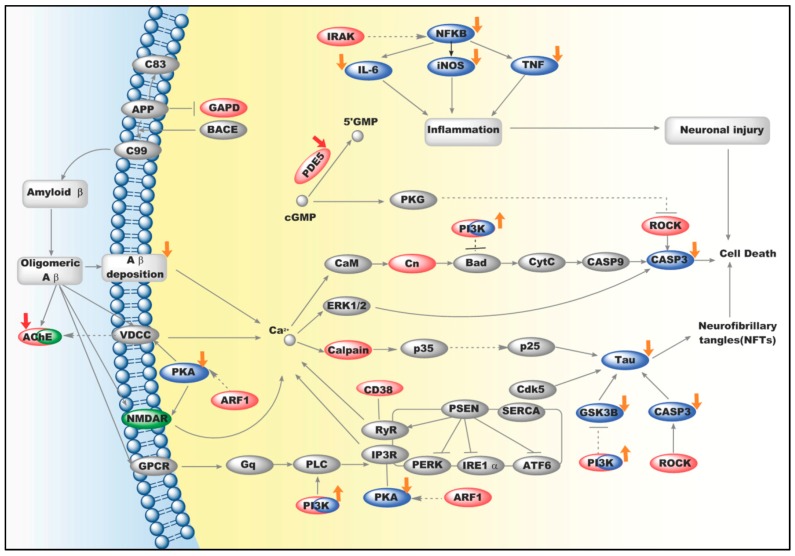
Icariin’s overall anti-Alzheimer's disease (AD) mechanistic network. Light Red ovals represent predicted icariin’s targets. Blue ovals represent indirectly regulated genes by icariin with experimental results. Yellow arrows represent indirect effect from icariin on these genes. Red arrows represent direct effect from icariin on these targets. The direction of arrows refers to icariin’s effects on targets (activate/upregulate or inhibit/downregulate). A green oval represents approved therapeutic target for AD.

To further explain the detailed mechanism of icariin, we built an integrative network based on both icariin’s targets and ADPs. Firstly, seven the Kyoto Encyclopedia of Genes and Genomes (KEGG) pathways (seen in Table 2) were significantly regulated by icariin’s putative targets. Then, we integrated eight of icariin’s putative targets which were involved in above pathways, together with two known icariin’s targets (AChE and PDE5), and “Alzheimer’s Disease Pathway” (defined in KEGG (has:05010)) into a network (shown in Figure 2). In the integrated network, the anti-AD mechanism of icariin may be inferred from three aspects: attenuation of hyperphosphorylation of tau protein, anti-inflammation and regulation of Ca^2+^ homeostasis.

**Table 2:** Significantly enriched pathways influenced by icariin’s targets.

KEGG Pathway	The Number of Icariin’s Targets	*p*-Value
Spliceosome	5	2.26 × 10^−6^
Vibrio cholerae infection	2	3.17 × 10^−2^
Carbohydrate digestion and absorption	3	7.34 × 10^−3^
Legionellosis	3	4.59 × 10^−2^
Oxytocin signaling pathway	5	4.25 × 10^−2^
cGMP-PKG signaling pathway	5	3.09 × 10^−2^
Apoptosis	5	1.14 × 10^−2^

At the early stage of the AD evolution, the tau protein can be hyperphosphorylated and then contribute to neurodegeneration [22]. Previous experimental results have demonstrated that icariin could lessen the extent of hyperphosphorylation of tau protein which was induced by Aβ. Meanwhile, icariin enhanced survival of neuronal cells by blocking excessive activation of GSK-3β [7]. In our network, icariin seemed to influence tau protein by targeting PI3K, as Figure 2 indicated. Actually, it was reported that the PI3K/Akt signaling pathway could be stimulated by icariin [8], and further the GSK-3β activity could be inhibited [23]. In addition, cleavage of tau by Caspase-3 (CASP3) may precede and lead to the formation of NFTs, which produce further permanent toxicity for neurons in the brains of patients with AD [24]. Our network indicated that icariin may interact directly with ROCK which would regulate CASP3. Previous experimental evidence suggested that icariin could reduce CASP3 activity [25].

Inflammation in neuronal cells is well known in AD progression [26,27]. Our study suggested that icariin may target IRAK, upstream elements of inflammatory cytokines, as shown in Figure 2. This agreed well with the report that icariin could downregulate NFkB [28] and inflammatory cytokines, such as TNF, iNOS and interleukins [29,30].

The calcium dysregulation plays an important role in AD pathogenesis and accompanies almost the whole brain pathologic process observed in AD patients [31]. Our results indicated that icariin may regulate cell Ca^2+^ through targeting ARF1, which was found to activate PKA pathway [32]. Meanwhile, the calcium permeability of NMDAR was reported to decline when PKA was inhibited [33]. As a well-known calcium influx, NMDAR is also a disease target of AD, where memantine was invented as an antagonist of NMDAR [3]. Since previous experimental results demonstrated that icariin could down-regulate PKA activity [34], we inferred that icariin may inhibit ARF1 activity leading to suppressing PKA activity, and further declining the calcium permeability of NMDAR.

Interestingly, AChE was successfully predicted as direct target of icariin, agreeing well with previous results [14]. Combining with the upstream effects of calcium regulation through voltage-dependent calcium channels (VDCC) [35], icariin might produce anti-AD effects in a synergistic way by acting on AChE both directly and indirectly, as Figure 2 indicated. Coincidentally, the synergistic effect also happened to PI3K. Icariin was reported to activate PI3K/Akt pathway through phosphorylation of Akt (Ser473) [7]. Meanwhile, we inferred that icariin might directly bind to PI3K and activate it as well. It seemed that, despite icariin’s synergistic effects on PI3K as well as AChE, further experimental validation for the binding status between icariin and PI3K was still required.

## 3. Discussion

In the present study, an inverse-docking technology was employed to predict icariin’s molecular targets to study the anti-AD mechanism. Then, a molecular network was constructed for systematic view of anti-AD mechanism by jointing predicted targets with known AD proteins. Finally, we found that attenuation of hyperphosphorylation of tau protein, anti-inflammation and regulation of Ca^2+^ homeostasis may contribute to the anti-AD effects of icariin.

As an *in silico* approach, INVDOCK is generally used to identify putative protein targets for small molecules based on physi-chemical complementarity between compounds and protein cavities. With the increasing accumulation of protein structures, INVDOCK has been widely applied to explore not only the therapeutic mechanism [36], but also the toxicity and side effects of a molecule [37]. In our study, we started searching within neurodegenerative disease-related proteins to identify putative targets for icariin. It is noted that INVDOCK could not differentiate between activation or inhibition effects of the compound. Thus, the putative targets may relate to not only therapeutic effects but also adverse or side effects. Through mapping to AD-related pathway and collecting literature support, the icariin’s effect on direct targets involving the integrated anti-AD pathway would be inferred by known upstream or downstream genes regulated by icariin. For example, although IRAK was predicted to be icariin’s target, the specific effect was undefined. It was reported that IRAK could activate its downstream genes such as NF-ΚB. Furthermore, these downstream gene expressions were decreased when icariin appeared. So it would be inferred that icariin might directly inhibit IRAK and then reduce inflammation. As another example, ARF1 was also predicted as icariin’s direct target and the effect is as yet undefined. Through the downstream genes, PKA downregulated by icariin, it was also inferred that icariin might primarily inhibit ARF1 leading to downregulation of PKA, further declining the calcium permeability of NMDAR, and finally resulting in Ca^2+^ homeostasis change. In addition, the direct activation of PI3K by icariin was suggested since icariin would decrease the expression of GSK-3β, which was downregulated by PI3K. Identification of putative targets, together with literature or experimental support, may help to better understand the anti-AD mechanism of icariin.

Previously, a systematical study was conducted by Sun *et al.* to study the anti-AD mechanism of four herbal medicines (Ginkgo biloba, Huperzia serrate, Melissa officinalis, Salvia officinalis) [17]. In the above paper, herbal ingredients were used as molecular probes to detect the AD pathogenesis where six pathways were mainly suggested: three disease-associated pathways: AD, cancer, and diabetes mellitus; the calcium ion signal transduction pathway; the inflammatory cytokine-associated pathway; and the cell proliferation pathway. Interestingly, in addition to Ca^2+^ homestasis and inflammatory cytokines, icariin seems to target tau protein formation, suggesting its promising potential in being further developing into successful anti-AD drugs. Herbal compounds have been regarded as an important library in drug discovery for a long time, while investigating the underlying molecular mechanism will help to modify or improve the compound activity in further being developed into better drugs. Icariin’s anti-AD mechanism was investigated *in silico* through ligand–protein docking strategy and systematically integrated network. With future experimental validation, the anti-AD targets are expected to provide assistance to optimize the specificity and activity of icariin’s derivatives. Similarly, the framework in this study would help to facilitate drug development from the herbal compound library.

## 4. Experimental Section

### 4.1. Identification of Putative Protein Targets

Neurodegenerative disease-related proteins were firstly retrieved from the Comparative Toxicogenomics Database (CTD) [38], and their cavity structures were obtained from the developed protein cavity database [18] which was derived from Protein Data Bank (PDB). INVDOCK, which was a flexible-docking software for finding potential protein targets of a small molecule, was used to screen against the above dataset for icariin. The icariin was pre-prepared by adding hydrogen, calculating the charge based on MMFF94x before target screening by the INVDOCK program. Then, each conformer of icariin, obtained by sampling, was aligned in the selected cavity depending on the position match between every atom of icariin and modeled center spheres. The conformation optimization based on molecular mechanics was performed by sampling rotatable bonds with the limitation of torsion space both for the ligand and for the side chain of protein located at binding sites. Meanwhile, limited side-chain conformation sampling of protein was allowed during energy minimization. The scoring of docked structures was calculated by a energy function of the ligand–receptor interaction, named as ΔE_LP_. It covered not only bonded hydrogen terms but also nonbonded terms in consideration of the following-up structure optimization. Here, two parameters (ΔE_Threshold_ and ΔE_Competitor_) were provided in the INVDOCK. We chose the default values as INVDOCK suggested. Finally, a neurodegenerative disease-related protein was considered as a putative target of icariin when the molecule would be docked into the protein and the binding score satisfied the criterion [39].

### 4.2. Average Shortest Path Calculation

**Figure 3:**
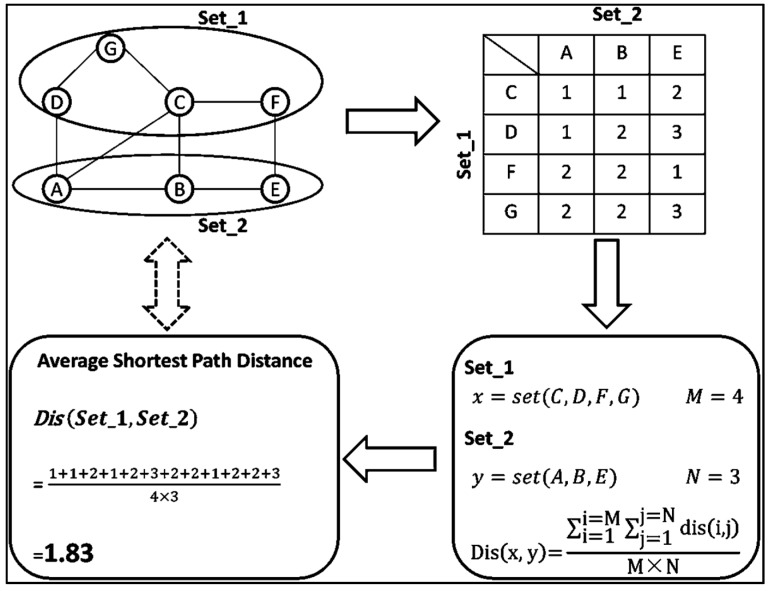
Average shortest path calculation of inter-subnetworks.

Average shortest path, to measure the performance of information transport in a network, refers to the averaged length of the shortest paths for all paired nodes [40]. This parameter was also applied on inter-subnetwork issue by calculating the average distance for all possible pairs of nodes from two subnetworks. As illustrated in Figure 3, given two subsets of genes, Set_1 (D, G, C, F) and Set_2 (A, B, E) in a background network, the shortest paths were calculated for all possible pairs between Set_1 and Set_2 [41]. The average shortest distance between Set_1(x) and Set_2(y) was defined as:

(1) $$\text{Dis}\left(x,\;y\right)=\frac{\sum_{i=1}^{i=M}\sum_{j=1}^{j=N}\text{dis}\left(i,j\right)}{M\times N}$$

where dis(i, j) was a distance of the shortest path between the ith gene from set x and the jth gene from set y. In this study, the background Protein-Protein Interaction (PPI) network was constructed based on an online database (HPRD) [42].

### 4.3. The Semantic Similarity of Gene Ontology (GO) Profiles

The Gene Ontology semantic similarity would provide the functional comparison of gene products [43,44]. On the tree of Gene Ontology, each gene was classified into different gene groups. And each gene group was named as a GO term according to the involved biological processes. Given two gene sets, each of them would be annotated as a profile of GO terms, in which are significantly enriched (*p*-value less than 0.05 in hypergeometric test). Firstly, the semantic similarity of two GO terms was computed by a graph-based strategy using the topology of the GO graph structure [45]. Then, the semantic similarity of two profiles of GO terms was computed based on the best-match average strategy. The two steps were employed by GOSemSim package from Bioconductor [46].

### 4.4. Pathway Enrichment of Icariin’s Putative Targets

Pathway enrichment analysis was used to determine whether a pathway was significantly regulated by icariin. Fisher's exact test was used to quantitatively measure whether a pathway was more enriched with icariin’s targets than would be expected by chance. These pathways with a *p*-value < 0.05 would be regarded as significantly regulated by icariin.

## Supplementary Materials

Supplementary materials can be found at http://www.mdpi.com/1422-0067/17/1/113/s1.
