# Supplementary material for: In Silico Insight into Potential Anti-Alzheimer’s Disease Mechanisms of Icariin
Source: Int J Mol Sci. 2016 Jan 15;17(1):113. doi: 10.3390/ijms17010113 (PMC4730354; doi:10.3390/ijms17010113)
Supplement: Supplementary file 1 [file ijms-17-00113-s001.zip › ijms-110822-Supplementary Materials/ijms-110822-supplementary tables.pdf]

Supplementary Materials: *In Silico* Insight into Potential Anti-Alzheimer's Disease Mechanisms of Icariin

Zhijie Cui, Zhen Sheng, Xinmiao Yan, Zhiwei Cao and Kailin Tang

Table S1. 59 potential targets of icariin predicted by INVDOCK.

Uniprot	Gene Symbol	Gene ID	Target Type
Q13464	ROCK1	6093	experimental
P00439	PAH	5053	approved
Q9HAN9	NMNAT1	64802	experimental
Q9BW91	NUDT9	53343	experimental
P50135	HNMT	3176	approved
Q10588	BST1	683	experimental
P06737	PYGL	5836	approved
O75874	IDH1	3417	experimental
P00750	PLAT	5327	approved
O76074	PDE5	8654	approved
P84077	ARF1	375	experimental
P63098	PPP3R1	5534	experimental
Q08209	PPP3CA	5530	experimental
P12821	ACE	1636	approved
P43490	NAMPT	10135	experimental
O75164	JMJD2A	9682	NA
P04040	CAT	847	approved
P06276	BCHE	590	approved
Q92871	PMM1	5372	NA
P04181	OAT	4942	approved
P13569	CFTR	1080	approved
P02774	GC	2638	approved
O14815	CAPN9	10753	NA
P11387	TOP1	7150	approved
P48736	PIK3CG	5294	experimental
Q16836	HADH	3033	experimental
Q92947	GCDH	2639	approved
P04062	GBA	2629	approved
Q9NWZ3	IRAK4	51135	experimental
P51795	CLCN5	1184	NA
Q8TBC4	UBA3	9039	NA
Q9Y5S9	RBM8A	9939	NA
Q9UKL6	PCTP	58488	experimental
Q96T66	NMNAT3	349565	experimental
P28907	CD38	952	NA
Q10471	GALNT2	2590	NA
P15291	B4GALT1	2683	approved
Q9UBT2	UBA2	10054	NA
P07737	PFN1	5216	experimental
Q07889	SOS1	6654	NA
P04406	GAPDH	2597	experimental

Table S1. *Cont.*

Uniprot	Gene Symbol	Gene ID	Target Type
P14902	INDO	3620	approved
P35080	PFN2	5217	experimental
P04183	TK1	7083	approved
P21399	ACO1	48	NA
P09012	SNRPA	6626	experimental
P14678	SNRPB	6628	NA
Q86SR1	GALNT10	55568	NA
P19367	HK1	3098	experimental
P19883	FST	10468	experimental
P08476	INHBA	3624	NA
P0DMV8	HSPA1A	3303	NA
P0DMV9	HSPA1B	3304	NA
P27695	APEX1	328	approved
P02686	MBP	4155	NA
P17655	CAPN2	824	NA
P52789	HK2	3099	NA
P22303	AChE	43	approved
P14902	INDO	3620	approved

Table S2. The binding affinity comparison between icariin and corresponding known ligand in same protein target (all).

Uniprot	Gene Symbol	PDB Code	Known Ligands			Icariin	
			Name	MM/GBVI (kcal/mol)	Affinity (pki)	MM/GBVI (kcal/mol)	Affinity (pki)
Q13464 *	ROCK1	2ETK	Hydroxyfasudil	−21.03	8.10	−33.02	14.34
P00439 *	PAH	4PAH	Norepinephrine	−26.24	7.05	−44.04	7.90
Q9HAN9 *	NMNAT1	1GZU	Nicotinamide Mononucleotide	−27.30	12.54	−23.15	17.75
Q9BW91 *	NUDT9	1Q33	β-D-Glucose	−24.64	10.70	−34.00	13.26
P50135 *	HNMT	2AOU	Amodiaquine	−26.05	6.91	−20.88	7.00
Q10588 *	BST1	1ISG	Adenosine-5′-diphosphate Monothiophosphate	−14.33	9.41	−24.31	10.99
P06737 *	PYGL	1FA9	Adenosine Monophosphate	−17.06	8.66	−27.18	9.79
O75874	IDH1	3MAP	Isocitric Acid	−23.70	11.44	−6.68	12.24
P00750 *	PLAT	1PK2	Aminocaproic Acid	−19.90	9.71	−24.92	9.30
O76074 *	PDE5	2H42	Sildenafil	−36.25	9.67	−28.87	13.89
P12821	ACE	2X92	Ramipril	−75.94	18.17	−28.89	12.78
P43490	NAMPT	2GVJ	*N*-[4-(1-Benzoylpiperidin-4-yl)butyl]-3-pyridin-3-ylpropanamide	−36.335	8.765	−1.330	10.173
P06276	BCHE	4BDS	Tacrine	−14.76	5.99	34.33	11.88
P04181	OAT	2CAN	Canaline	−31.83	17.52	−20.51	14.68
P48736	PIK3CG	1E8Z	Staurosporine	−20.34	10.29	19.19	18.11
Q16836	HADH	1F17	NADH	−56.41	15.93	−19.99	11.56
Q92947	GCDH	1SIQ	Flavin adenine dinucleotide	−51.45	11.24	−31.74	11.81
P04062 *	GBA	2F61	2-(Acetylamino)-2-deoxy-a-D-glucopyranose	−16.41	5.57	−21.80	11.17
Q9NWZ3	IRAK4	2NRU	1-(3-Hydroxypropyl)-2-[(3-nitrobenzoyl)amino]-1h-benzimidazol-5-yl Pivalate	−38.29	9.69	−15.97	13.96
Q9UKL6	PCTP	1LN3	Palmitoyl-Linoleoyl Phosphatidylcholine	−70.20	15.41	−14.06	14.00
Q96T66	NMNAT3	1NUT	A,β-Methyleneadenosine-5′-triphosphate	−78.12	16.31	−37.47	15.35
P15291 *	B4GALT1	4EEG	*N*-Acetyl-D-glucosamine	−19.15	8.97	−34.21	10.75
P07737 *	PFN1	1CJF	7-Hydroxy-4-methyl-3-(2-hydroxy-ethyl)coumarin	−15.42	6.61	−27.05	8.67
P35080	PFN2	1D1J	1-Methoxy-2-[2-(2-methoxy-ethoxy)]-ethane	−8.23	3.24	52.45	14.16
P04183	TK1	1XBT	Trifluridine	−181.66	27.76	35.21	13.53
P09012 *	SNRPA	1NU4	Malonic acid	−22.16	6.52	−23.86	5.34
P19367	HK1	1CZA	α-D-Glucose-6-phosphate	−62.52	15.02	−14.46	10.65
P84077 *	ARF1	1U81	1,3-Propandiol	−37.77	4.64	−43.17	11.18
P63098	PPP3R1	1MF8	ISA247	−45.73	14.07	−30.17	15.09
Q08209 *	PPP3CA	4F0Z	Myristic acid	−15.56	4.64	−22.51	8.90
P04040	CAT	1DGH	Fomepizole	−9.18	4.72	0.86	17.26
P13569 *	CFTR	2BBO	Ibuprofen	−8.23	6.00	−6.62	11.014

Table S2. *Cont*.

Uniprot	Gene Symbol	PDB Code	Known Ligands			Icariin	
			Name	MM/GBVI (kcal/mol)	Affinity (pki)	MM/GBVI (kcal/mol)	Affinity (pki)
P02774 *	GC	1J78	Cholecalciferol	−10.99	5.05	−21.56	6.43
P11387 *	TOP1	1TL8	Irinotecan	−34.75	14.32	−20.04	17.80
P04406	GAPDH	1U8F	Thionicotinamide-Adenine-Dinucleotide	−47.00	8.60	−25.29	8.29
P14902	INDO	4PK5	Melatonin	−7.34	6.32	−1.35	15.63
P19883 *	FST	2B0U	D-Myo-Inositol-Hexasulphate	−9.54	7.03	−24.81	9.78
P27695 *	APEX1	4QHE	Lucanthone	−16.70	4.25	−17.73	4.56
P22303 *	AChE	1F8U	Mefloquine	−11.44	6.54	−34.86	7.97

For each therapeutic target, same active pocket site was adopted for binding affinity comparison between icariin and known ligands. The docking was demonstrated and refined using the software MOE with the parameters of Receptor: Receptor + Solvent; Placement: Triangle Mather; Rescoring 1: London dG, Retain: 30; Refinement: Forcefield (MMFF94x); Rescoring 2: London dG, Retain: 30. Targets (Uniprot ID) labeled by "*" are regarded as strong or true effect by icariin since icariin-target interactions show comparative binding affinities (better or close MM/GBVI or pki value).
